# Supplementary material for: Effect of acid-suppressive drugs on endoscopic transmural drainage of peripancreatic fluid collections—a randomized controlled trial
Source: PeerJ. 2025 Aug 19;13:e19872. doi: 10.7717/peerj.19872 (PMC12372783; doi:10.7717/peerj.19872)
Supplement: Supplemental Information 2 [file peerj-13-19872-s002.docx]

**Project summary**

This study was a randomized controlled trial of the effects of acid-suppressive drugs on endoscopic drainage of peripancreatic effusion and on microbiotes in peripancreatic effusion. The experimental group and the control group were randomly divided, the experimental group was given acid-inhibiting drugs, and the control group was not given acid-inhibiting drugs. The difference of microorganisms in cystic fluid and the related indicators of complications after endoscopic drainage were analyzed, so as to determine the influence of acid-suppressive drugs on peripancreatic hydrops after endoscopic drainage and the influence of microorganisms in peripancreatic hydrops.

**General information**

The study came from a large tertiary endoscopic clinic in China. The research was supported by the Natural Science Foundation of Hebei Province, China. The principal person in charge is Dr. Zhang Lichao.

**Rationale & background information**

Peripancreatic fluid collections(PFCs) often occur as a result of acute or chronic pancreatitis or pancreatic injury. Multiple studies have demonstrated that endoscopic drainage offers numerous advantages in terms of efficacy, safety, and patient acceptance, and has become a first-line treatment option.

Acid suppressive drugs, especially proton pump inhibitors, are widely used in patients with acute pancreatitis worldwide. Proton pump inhibitors inhibit gastric acid secretion by inhibiting H+-K+-ATPase, thereby indirectly inhibiting pancreatic secretion, and can prevent the occurrence of stress ulcer. Therefore, many physicians administer acid-suppressing drugs during endoscopic transmural drainage of peripancreatic fluid to reduce the occurrence of bleeding, ulcers, and other complications. Acid suppressant drugs can change the microbial composition of digestive tract by inhibiting the secretion of gastric acid. At the same time, previous studies have suggested that duodenal microbes are highly similar to pancreatic microbes. However, it remains unclear whether this has an impact on the microbiota of pancreatic fluid, thereby affecting clinical infections. Currently, there is no consensus on the use of acid-suppressing drugs during the perioperative period of endoscopic drainage.

**Study goals and objectives**

The aim of this study is to investigate the effects of preoperative acid-suppressing drug use in endoscopic transmural drainage of peripancreatic fluid through a randomized controlled trial. Specifically, we aim to assess the impact on the microbiota of pancreatic fluid and postoperative infections, providing a theoretical basis for the clinical application of acid-suppressing drugs.

**Study design**

### This study was designed as a randomized, placebo-controlled, double-blind exploratory trial aiming to investigate the role of acid-inhibiting drug in patients with non-infected peripancreatic fluid collections undergoing EUS-guided transmural drainage of Peripancreatic ﬂuid collections from a microbial perspective. Following ethical approval from our medical center's institutional review board, consecutive recruitment of patients with peripancreatic fluid collections was conducted in the department of biliary pancreas endoscopy surgery. Written informed consent was obtained from all patients prior to enrollment. All patients had complete access to all data collected in the study.

### Selection Criteria

### Inclusion criteria:

### 1. Patients with a CT or MRI diagnosis of peripancreatic fluid formation.

### 2. Patients with indications for endoscopic drainage, including pain, nausea, and jaundice, but not due to infection.

### 3. Fluid accumulation persisting for more than 6 weeks and unresponsive to conservative treatment.

### Exclusion criteria:

### 1. Patients with spontaneous infection or spontaneous hemorrhage within the cyst.

### 2. Patients with contraindications such as portal hypertension and gastrointestinal bleeding.

### 3. Patients who have received antibiotics within the past month.

### 4. Patients receiving acid-suppressing drugs due to peptic ulcer or other reasons.

### Study Groups

### After meeting the inclusion and exclusion criteria, patients in the study group were randomly allocated to either the acid inhibition group (group1) or the non-acid inhibition group (group2). The statistician provided computer-generated randomization assignments using a block randomization method. The random assignments were placed in opaque envelopes and impartially assigned in a 1:1 ratio between the two groups by the ward residents, who were the only individuals with access to the blinded data.

### In the acid-suppressive group, patients received acid-suppressive medication (omeprazole 80mg intravenously) 7 days before endoscopic drainage. In the non-acid inhibition group, patients received a placebo (intravenous saline administration) starting 7 days before the endoscopic transmural drainage procedure. If a patient presented with symptoms suggestive of infection prior to surgery, antibiotics were administered to ensure patient safety, leading to exclusion from the study. Eventually, a total of 45 patients were enrolled in the study, including 25 patients in the acid inhibition group and 20 patients in the non-acid inhibition group.

### Procedure and sample collection

### All procedures were performed by the same experienced endoscopist. Intravenous anesthesia was administered to all patients. The entire operation process adhered to strict aseptic techniques, with all experiments and surgical materials undergoing rigorous sterilization procedures. Endoscopic ultrasound (OLYMPUS, JAPAN) was utilized for precise pancreatic scanning, ensuring exclusion of bleeding and solid nodules. Subsequently, the cyst was punctured using an ECHO-19 puncture needle (COOK) at a suitable location (either the stomach or duodenum), avoiding blood vessels. Once confirmation of needle entry into the cyst was achieved, 5-10ml of cyst fluid was extracted using a sterile negative pressure syringe. The collected fluid was then placed in a sterile test tube and promptly stored in a -80℃ refrigerator for subsequent microbiological analysis. Following the puncture, a guide wire was inserted to facilitate cystotomy and channel dilation. Depending on the patient's specific cyst condition, a plastic or metal stent was inserted during the procedure(The procedure was determined by an experienced digestive endoscopist, and this did not affect the study results).

### Methodology

### After meeting the inclusion and exclusion criteria, patients in the study group were randomly allocated to either the acid inhibition group (group1) or the non-acid inhibition group (group2). The statistician provided computer-generated randomization assignments using a block randomization method. The random assignments were placed in opaque envelopes and impartially assigned in a 1:1 ratio between the two groups by the ward residents, who were the only individuals with access to the blinded data.

### In the acid-suppressive group, patients received acid-suppressive medication (omeprazole 80mg intravenously) 7 days before endoscopic drainage. In the non-acid inhibition group, patients received a placebo (intravenous saline administration) starting 7 days before the endoscopic transmural drainage procedure. If a patient presented with symptoms suggestive of infection prior to surgery, antibiotics were administered to ensure patient safety, leading to exclusion from the study. Eventually, a total of 45 patients were enrolled in the study, including 25 patients in the acid inhibition group and 20 patients in the non-acid inhibition group.

### Procedure and sample collection

### All procedures were performed by the same experienced endoscopist. Intravenous anesthesia was administered to all patients. The entire operation process adhered to strict aseptic techniques, with all experiments and surgical materials undergoing rigorous sterilization procedures. Endoscopic ultrasound (OLYMPUS, JAPAN) was utilized for precise pancreatic scanning (Fig1), ensuring exclusion of bleeding and solid nodules. Subsequently, the cyst was punctured using an ECHO-19 puncture needle (COOK) at a suitable location (either the stomach or duodenum), avoiding blood vessels. Once confirmation of needle entry into the cyst was achieved, 5-10ml of cyst fluid was extracted using a sterile negative pressure syringe. The collected fluid was then placed in a sterile test tube and promptly stored in a -80℃ refrigerator for subsequent microbiological analysis. Following the puncture, a guide wire was inserted to facilitate cystotomy and channel dilation. Depending on the patient's specific cyst condition, a plastic or metal stent was inserted during the procedure(The procedure was determined by an experienced digestive endoscopist, and this did not affect the study results).

### Definition

### Technical success was defined as the successful placement of the stent into the cyst cavity under the guidance of an endoscope and radiography, with no displacement occuring. Clinical success was defined as complete resolution of the effusion or a maximum effusion diameter < 2cm, along with the absence of any clinical symptoms. Recurrence referred to the reappearance of fluid accumulation around the pancreas or clinical symptoms occurring 6 months after successful endoscopic drainage. Reoccurrence of effusion within six months is clinical treatment failure and is not defined as recurrence. Bleeding was determined by the presence of hematemesis, hematochezia, or a decline in hemoglobin of > 10g/L within 72 hours post-surgery. As long as there is a body temperature greater than 38.5 degrees Celsius after surgery, regardless of whether there is an increase in white blood cells, it is defined as infection. Once infection is confirmed, treatment is given immediately with intravenous antibiotics (ceftriaxone 2g, intravenous). The primary outcome measure focused on differences in microbial species and abundance between the two groups, with clinical infection, white blood cell count, and C-reactive protein were secondary outcomes.

### Safety considerations

### Acid suppressive drugs are widely used in clinical practice and the incidence of adverse events is very low. At the same time, patients had liver and kidney function during treatment. Once drug-induced loss was found, the experiment was stopped immediately and corresponding treatment was given

### Follow-up

### Patients were followed up for six months to one year. The main follow-up after discharge was recurrence of peripancreatic effusion

### Data management and statistical analysis

### The main outcome of our study was to observe the difference of microbiota in the two groups of cyst fluid. The sample size calculation is based on 16S microbial high-throughput sequencing, that is, when there are > 4 samples per group, it can be analyzed and produce meaningful results. In order to improve the credibility of the study, we defined the sample size as 45 cases. Statistical analyses were performed using IBM SPSS 27.0 and R 3.4.4 with a test level of α = 0.05. T-tests (continuous variables) and Chi-square tests (categorical variables) were used to assess inter-group differences. Shannon index, Simpson index,Chao1 index and observed species index were used to analyze the α diversity between the two groups. The differences in the Alpha diversity between the groups were analysed using the Kruskal–Wallis rank sum test. The Adonis method was used to analyze the differences in beta diversity between groups.

### Quality assurance

In the process of this study, we have professional clinicians to conduct operations and professional nursing teams to collect and preserve specimens. At the same time, we have professional biologists and statisticians to provide quality assurance for subsequent sac fluid analysis.

### Expected outcomes of the study

### The expected result of this study was to confirm the effect of peripancreatic effusion on the microbiome of the cyst fluid. Whether it is positive or negative, it provides a basic theoretical basis for the clinical use of acid suppressive drugs in patients with peripancreatic effusion, and lays a foundation for subsequent research.

### Dissemination of results and publication policy

### The study will be published and the data shared with the whole society after publication..

### Duration of the project

### August 2021 - August 2023:

### The subjects were subjected to corresponding operation methods and corresponding treatment, and samples were extracted.

### (1) Select research objects, collect patient data, and evaluate the status of patients. According to the experimental design, the study subjects were randomly divided into three groups, and the internal drainage of pancreatic pseudocyst was performed according to the conditions, and proton pump inhibitor and equal amount of normal saline were given in perioperative period.

### (2) The cyst drainage fluid was extracted and properly stored according to the time node, and the sample was sent to bacterial culture, along with bacterial DNA and high throughput

### Sequencing analysis

### (3) Collect records.

### August 2023 - March 2024:

### Analyze the research data and experimental results and write the paper.

### Problems anticipated

### The expected problems mainly include the small number of eligible patients and the inability to meet the sample size requirement. We extended the experiment time to 2 years to obtain enough samples

### Project management

### Yaoting Li: Data curation; methodology; formal analysis; writing – original draft. Tingting Yu and Senlin Hou: methodology; formal analysis.

### Wei zhang and Haiming Du: Conceptualization.

### Yankun Hou and Jiao Tian: data curation

### Lichao Zhang: Conceptualization; writing – review and editing.

### Ethics

### The study was approved by the Ethics Committee of the Second Hospital of Hebei Medical University and the Ethics Committee of the China Clinical Trial Center

### Informed consent forms

Each patient signed an informed consent prior to the trial.
